# An evaluation of the efficacy of a four-grade fat infiltration classification method, presented for the first time in literature

**DOI:** 10.1186/s12891-022-05180-5

**Published:** 2022-03-08

**Authors:** Alikemal Yazici, Tuba Yerlikaya, Adile Oniz

**Affiliations:** 1 Faculty of Medicine, Orthopedics and Traumatology Department, Near East University, Nicosia, Cyprus; 2 Orthopedics and Traumatology Department, Buyuk Anadolu Hospital, Samsun, Turkey; 3 Faculty of Health Sciences, Physiotherapy and Rehabilitation Department, Near East University, Nicosia, Cyprus; 4 Faculty of Health Sciences, Near East University, Nicosia, Cyprus; 5 Institute of Graduate Studies, Department of Biophysics, Near East University, Nicosia, Cyprus

**Keywords:** Paraspinal muscles, Intervertebral disc degeneration, Magnetic resonance imaging, Low back pain, Adipose tissue

## Abstract

**Background:**

The deficiency of the commonly used 3-grade fat infiltration system is discussed by comparing it with the 4-grade fat infiltration system, newly presented to the literature. The aim of this study was to evaluate the efficacy of a semi-quantitative simplified 4-grade fat infiltration measurement system, described for the first time in literature, through comparison with the existing simplified 3-grade fat infiltration system in the prediction of lumbar disc herniation.

**Methods:**

The study included 51 patients diagnosed with lumbar disc hernia and 50 healthy individuals as the control group. The patients were evaluated in respect of fat infiltration of the right and left lumbar multifidus and erector spina muscles on axial magnetic resonance imaging slices passing through the centre of the disc at L3-S1 level using the 3 and 4-grade fat infiltration measurement systems.

**Results:**

The 3-grade fat infiltration system was found to be insufficient in the prediction of lumbar disc herniation (*p* > 0.05) and the 4-grade fat infiltration system was determined to be effective in the prediction of lumbar disc herniation (*p* < 0.001).

**Conclusion:**

The 4-grade fat infiltration system was seen to be more effective than the 3-grade fat infiltration system in the determination of the level of fat infiltration in the paraspinal muscles and the prediction of lumbar disc herniation. The 4-grade fat infiltration system is a more effective semi-quantitative grading system which can be used instead of the simplified 3-grade system.

## Background

Lumbar disc herniation (LDH) is a common disease of the lumbar spine and a frequent cause of back pain, muscle spasms, and restricted movement [1–3]. The paraspinal muscles play a role in the functional and structural stabilisation of the lumbar spine. The outer layer, which is thought to be primarily responsible for spinal and extremity movements, is formed of the larger surface back muscles. The inner layer is a layer of deep muscle and primarily controls intersegmental movement [4].

Several methods are used to measure and evaluate fat infiltration of the paraspinal muscles, including several non-invasive methods such as computed tomography (CT), magnetic resonance imaging (MRI), ultrasound (US), magnetic resonance spectroscopy, chemical shift MRI, and multinuclear MRI [4]. Evaluation of fat infiltration can be made qualitatively, semi-quantitatively, and quantitatively [4]. MRI provides a clear image with high contrast and high resolution for soft tissue without radiation exposure. Moreover, the reliability of the results has been stated to be better than those of CT [5]. Therefore, it has become more preferred in recent years [6, 7].

Qualitative evaluation of muscles was first shown on CR scans by Goutallier et al. in a 5-grade visual spectrum of fat tissue infiltration of the rotator cuff muscle system. With developments in MRI, Fuchs et al. compared rotator cuff fat infiltration with CT and MRI evaluations [8, 9]. The 5-grade classification stated by Goutallier et al. was reduced to a 3-grade classification of normal, moderate, and severe. A simplified system was recommended by Slabough et al. [10]. Using standard criteria in adults, Solgaard Sorenson et al. visually classified fat infiltration as normal, mild, or severe, according to involvement at one or more lumbar levels [11]. Parkkola stated visual fat infiltration in 4 grades as normal, mild, significant, and severe [12]. Then by adding a percentage to this visual evaluation, it became semi-quantitative and was stated as a simplified 3-grade system evaluation by various authors [13–16]. More recently, fat infiltration of the paraspinal muscles has started to be measured quantitatively [17–24]. Goutallier's 5-grade classification is still widely used, especially in the evaluation of shoulder rotator cuff degeneration.

In the widely used semi-quantitative evaluation of fat infiltration, the 3-grade (< 10%, 10%-50%, > 50%) fat infiltration system is used [13–16]. However, in this grading system, 15% and 45% fat infiltration are included in the same percentage slice (10%-50%). This creates a weakness, and starting from the hypothesis that this could be insufficient to reach detailed results, a new alternative measurement method is here presented of 4 grades of fat infiltration (< 10%, 10%-30%, 30%-50%, > 50%), and the sensitivity of this system was evaluated. To confirm this hypothesis, the aim of the current study was to compare and evaluate the efficacy of the 3 and 4-grade fat infiltration measurement systems in the prediction of LDH.

## Material and method

### Subjects

This prospective observational study included 51 patients who presented at the Orthopaedics and Traumatology Department of Büyük Anadolu Hospital between November 2020 and February 2021 with the complaint of low back pain and were diagnosed with LDH. A control group was formed of 50 healthy subjects, selected at random from those invited to participate via social media and announcements, who had not experienced any low back pain in the last year and were not determined with any lumbar problem in physical or radiology examinations. The LDH patient group included patients aged < 65 years, with low back pain ongoing for the last 3 months, who were diagnosed with LDH on MRI. For patients with suspected root compression, EMG was requested, and after confirmation of no root compression, these patients were included in the study.

Patients were excluded from the study if radiculopathy was determined on MRI or EMG, if they had any rheumatological or infectious disease, any spine or hip deformity, a history of lumbar surgery, or acute pain in any other part of the body.

Demographic data and disease-related information were recorded on a demographic information form in face-to-face interviews. When necessary for the diffierential diagnosis, hemogram, erythrocyte sedimentation rate, full urine analysis, Anti-Streptolysin O (ASO), C-Reactive-Protein (CRP), Rheumatoid Factor (RF), salmonella, and brucella tests were requested. The physical examinations of 101 patients were performed by the same orthopaedics and traumatology specialist, experienced in spinal surgery, and the MRIs were analyzed by the same radiology specialist (K.T.) experienced on this subject and blinded to the clinical history. All the MRIs were taken by the same radiology technician. Approval for the study was granted by the Local Ethics Committee (YDU/2020/83–1160). Informed consent was obtained from all the study participants.

### Measurements

#### Magnetic resonance imaging

Images were obtained using a 1.5 Tesla MRI unit (Sigma Explorer SV25.3 with up-to-date software and 16 channels; General Electric, Milwaukee, WI, USA). With the patient in a supine position, a pillow was placed under the knees and the coil was placed on the spine. The measurements were taken with a routine protocol applied to the lumbar spine to pass through the centre of the disc at the measurement level between L3-S1 (L3-4/L4-5/L5-S1) without leaning to the right or left. Turbo-spin echo T1 and T2-weighted sagittal slices and turbo-spin echo T2 axial slices parallel to the disc spaces were obtained at a thickness of 4 mm. Evaluations were made on the T2 axial slices. Fat content was evaluated at all 3 levels of L3-S1 of the left and right erector spina (m. iliocostalis + m. longissimus) and the m. multifidus.

**Fig. 1:**
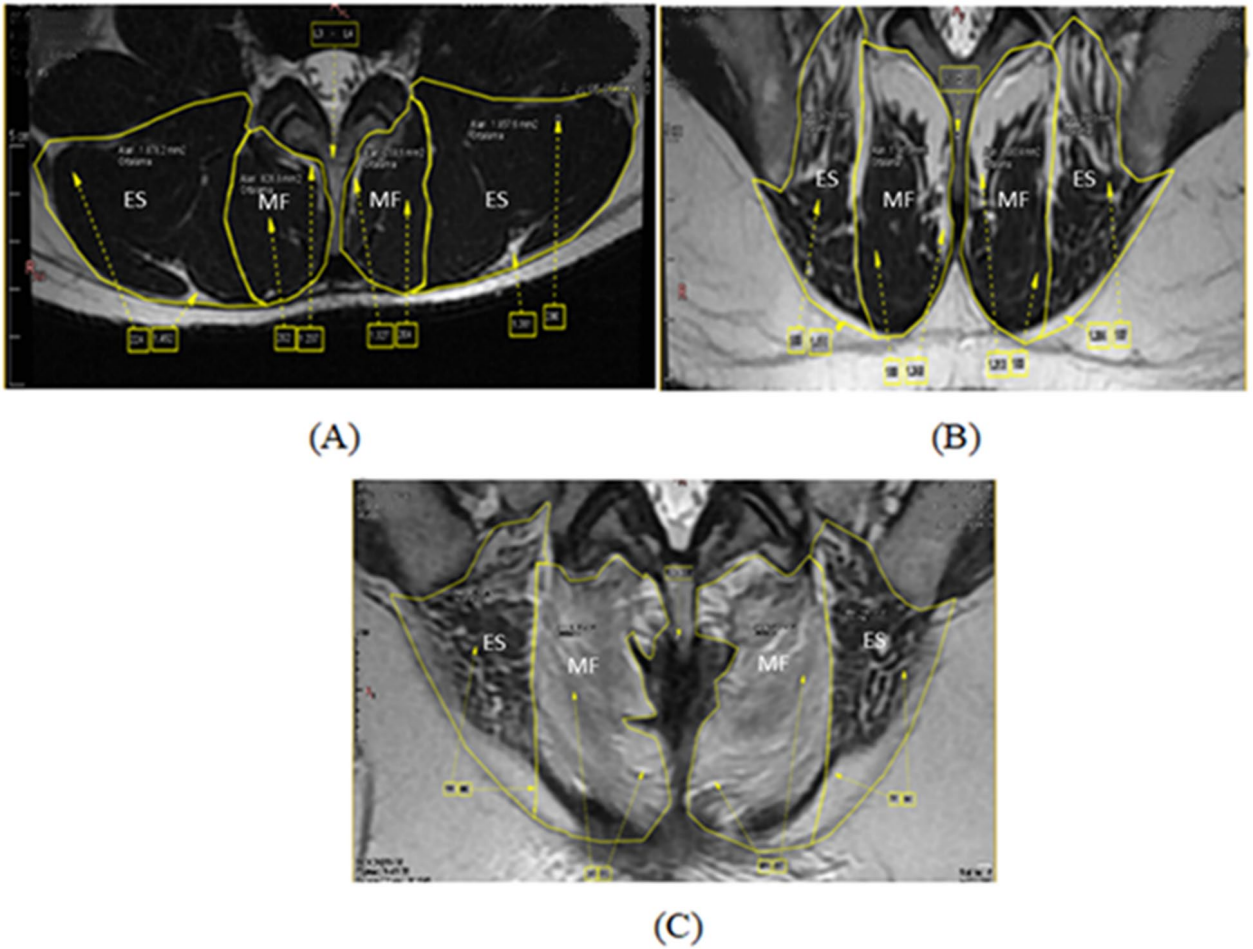
T2 axial magnetic resonance imaging slices showing location of the muscles and degree of fat infiltration, evaluated with the simplified 3-grade system. **A** Grade 1: < 10% fat infiltration, **B** Grade 2: 10%- 50% fat infiltration, **C** Grade 3: > 50% fat infiltration, Musculus multifidus (MF), Musculus erector spinae (ES)

**Fig. 2:**
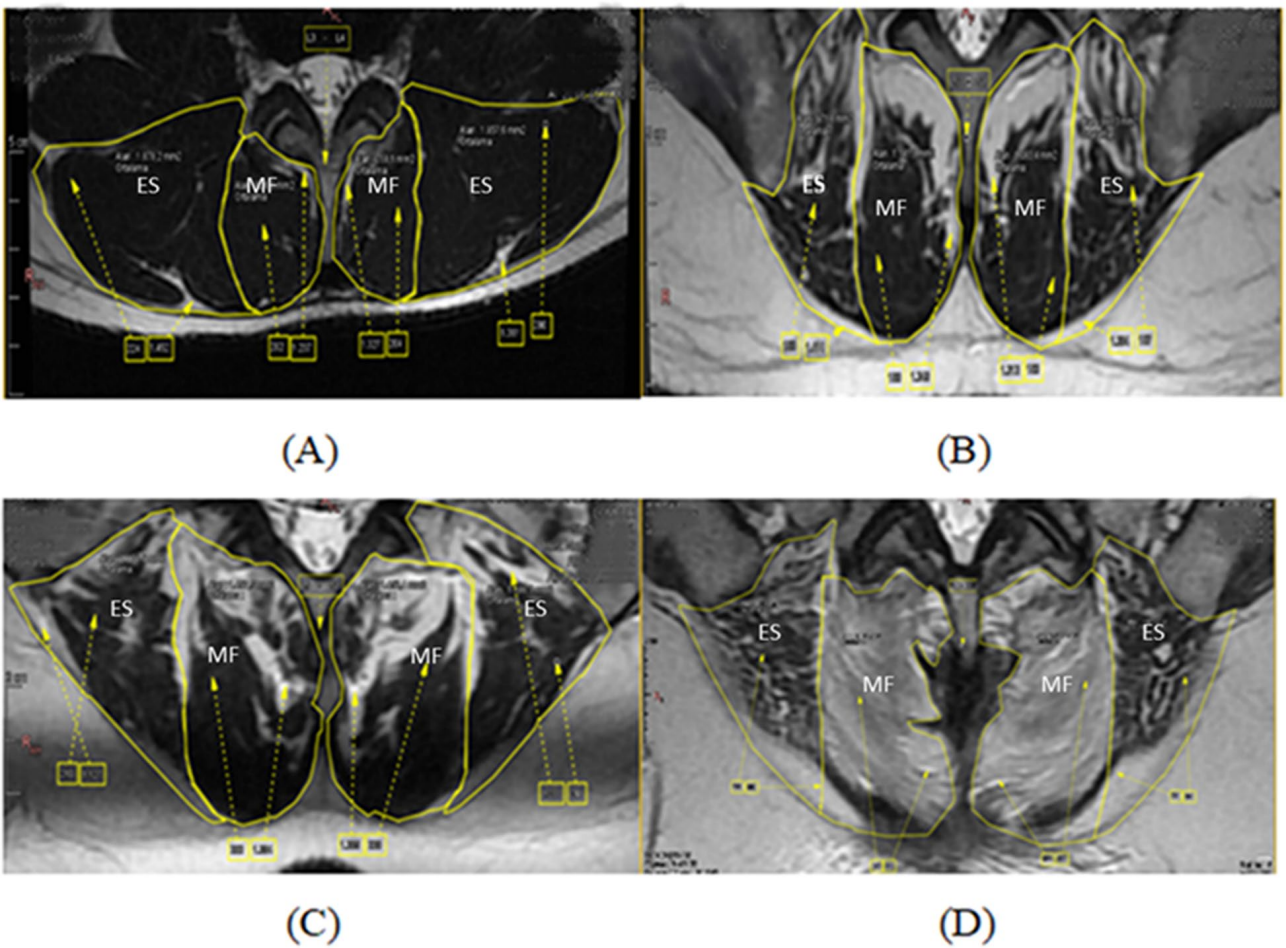
T2 axial magnetic resonance imaging slices showing location of the muscles and degree of fat infiltration, evaluated with the 4-grade system. **A** Grade 1: < 10% fat infiltration, **B** Grade 2: 10%- 30% fat infiltration, **C** Grade 3: 30%-50% fat infiltration, **D** Grade 4: > 50% fat infiltration, Musculus multifidus (MF), Musculus erector spinae (ES)

Fat infiltration of the muscles was evaluated semi-quantitatively, using the simplified 3-grade system and the 4-grade system. In the simplified 3-grade system, fat infiltration is graded as Grade 1: normal (fat infiltration of up to 10% of the muscle cross-sectional area [CSA]), Grade 2: moderate (10%-50%) and Grade 3: severe (> 50%) (Fig. 1) [2]. In the simplified 4-grade system, fat infiltration is graded as Grade 1: normal (fat infiltration of up to 10% of the muscle CSA), Grade 2: mild (10%-30%), Grade 3: moderate (30%-50%), and Grade 4: severe (> 50%) (Fig. 2).

For intra and inter-observer reliability, 20 randomly selected patients were re-evaluated one month later by the same radiologist (K.T) and a different radiologist (H.E). When the intra-observer agreement was examined with kappa, the kappa value was found to be 0.916 for the simplified 3-grade system and 0.909 for the 4-grade system. Inter-observer agreement was found to be 0.905 for the 3-grade system and 0.900 for the 4-grade system.

### Statistical evaluation

The study data were analyzed statistically using IBM SPSS vn. 23 software. In the comparisons of the age and BMI values between the groups, the Mann Whitney U-test was used. The Continuity Correction test was used in the examination of gender distribution. Binary logistic regression analysis was applied to the risk factors affecting LDH, and the results were presented as Odds Ratio (OR) (95% CI). In the univariate analysis, each variable was added separately to the model and the effect of each variable was examined alone. In the multivariate analysis, all the variables were placed in the model at the same time and the effects were examined together. A value of *p* < 0.05 was accepted as the level of statistical significance. According to the PostHoc Power analysis, the minimum sample number was determined to be 50 subjects when 95% confidence (1-α), 95% test power (1-β), and 0.32—0.80 degeneration values were taken into consideration. The study was completed with a total of 101 subjects to account for possible patient loss. The test power was determined to be 99.9% with 101 subjects according to the PostHoc Power analysis [25].

## Results

The demographic characteristics of the study participants according to groups are shown in Table 1. No difference was observed between the groups in respect of age, gender, and BMI (*p* = 0.205, *p* = 0.726, *p* = 1.000).

**Table 1:** Demographic data of the study groups

	Healthy Control group		LDH group		Total		
	$\overline{\mathrm{x} }$± σ	Median (min–max)	$\overline{\mathrm{x} }$± σ	Median (min–max)	$\overline{\mathrm{x} }$± σ	Median (min–max)	*P*
Age (years)	36.58 ± 10.32	36 (21—64)	37.43 ± 9.54	39 (20—51)	37.01 ± 9.89	38 (20—64)	0.205^1^
BMI (kg/m^2^)	25.76 ± 3.08	25.72 (19.25 – 32.79)	26.15 ± 4.66	25.95 (17.71 – 36.2)	25.95 ± 3.94	25.88 (17.71 – 36.2)	0.726^1^
Duration of LBP (months)		–-	37.63 ± 45.09	24 (3—240)			
VAS resting			2.29 ± 1.22	2 (1—6)			
VAS activity			4.22 ± 1.71	4 (2—8)			
Gender	n (%)		n (%)				
* Male*	22 (44.0)		23 (45.1)		45 (44.6)		1.000^2^
* Female*	28 (56.0)		28 (54.9)		56 (55.4)		

^1^Mann Whitney U, ^2^ Continuity Correction

### 3 Grade fat infiltration classification system

When fat infiltration of the parapinal muscles of the healthy control group and the LDH group was examined according to the simplified 3-grade system, the age, gender, BMI, right and left musculus multifidus (M.MF) and right and left musculus erector spinae (M. ES) values were not determined to be risk factors (*p* > 0.05) (Table 2).

**Table 2:** Logistic regression analysis results of risk factors affecting lumbar disc herniation with the simplified 3-grade fat infiltration system

	Univariate		Multivariate	
	OR (%95 CI)	*P*	OR (%95 CI)	*p*
Gender	1.045 (0.665–1.645)	0.848	1.186 (0.727 – 1.935)	0.496
Age (years)	1.009 (0.986–1.032)	0.452	1.004 (0.974 – 1.035)	0.795
BMI (kg/m^2^)	1.026 (0.968–1.086)	0.389	1.027 (0.952 – 1.108)	0.498
Right M.MF*	1.18 (0.834–2.082)	0.238	1.04 (0.499 – 2.167)	0.916
Left M.MF*	1.421 (0.904–2.236)	0.128	1.665 (0.8 – 3.464)	0.173
Right M.ES*	1.104 (0.713–1.708)	0.658	1.307 (0.524 – 3.259)	0.566
Left M.ES*	1.002 (0.642–1.563)	0.993	0.606 (0.237 – 1.55)	0.296

The bands of < 10%; 10%-50%, > 50% were used in the classification, *M.MF* Musculus multifidus, *M.ES* Musculus erector spinae

### 4 Grade fat infiltration classification system

In the examination of the fat infiltration of the healthy control group and the LDH group according to the 4-grade system, when right M.MF < 10% was taken as reference as a result of the univariate analysis, in those with fat infiltration rate of 10–30%, the risk of LDH was 2.914-fold greater up to 5.291-fold (*p* < 0.001) (Table 3). Similarly, in the multivariate analysis when the right M.MF value of < 10% was taken as reference, in those with fat infiltration rate of 10–30%, the risk of LDH was 5.806-fold greater up to 16.359-fold (*p* = 0.001). When left M.ES < 10% was taken as reference as a result of the univariate analysis, in those with fat infiltration rate of 10–30%, the risk of LDH was 1.903-fold greater up to 3.301-fold (*p* = 0.022), and in those with 30%-50% fat infiltration, the risk was 3.578-fold greater increasing to 8.716-fold (*p* = 0.005).

**Table 3:** Logistic regression analysis results of risk factors affecting lumbar disc herniation with the simplified 4-grade fat infiltration system

	Univariate		Multivariate	
	OR (%95 CI)	*p*	OR (%95 CI)	*P*
Gender	1.045 (0.665–1.645)	0.848	1.440 (0.844 – 2.455)	0.181
Age (years)	1.009 (0.986–1.032)	0.452	1.005 (0.973 – 1.038)	0.779
BMI (kg/m^2^)	1.026 (0.968–1.086)	0.389	1.031 (0.951 – 1.119)	0.457
Right M.MF* (reference: < %10)				
* %10-%30*	2.914 (1.605–5.291)	** < 0.001**	5.806 (2.061 – 16.359)	**0.001**
* %30-%50*	1.478 (0.581–3.758)	0.412	3.195 (0.362 – 28.226)	0.296
Left M.MF* (reference: < %10)				
* %10-%30*	1.401 (0.810–2.419)	0.227	0.373 (0.138 – 1.011)	0.053
* %30-%50*	0.876 (0.342–2.241)	0.782	0.226 (0.023 – 2.257)	0.205
Right M.ES* (reference: < %10)				
* %10-%30*	1.345 (0.788–2.297)	0.278	0.330 (0.100 – 1.092)	0.070
* %30-%50*	1.373 (0.629–2.997)	0.427	0.110 (0.022 – 0.549)	**0.007**
Left M.ES* (reference: < %10)				
* %10-%30*	1.903 (1.097–3.301)	**0.022**	4.164 (1.230 – 14.097)	**0.022**
* %30-%50*	3.578 (1.469–8.716)	**0.005**	36.99 (5.700 – 240.062)	** < 0.001**

^*^The bands of < 10%; 10%-30%, 30%-50%, > 50% were used in the classification, *M.MF* Musculus multifidus, *M.ES* Musculus erector spinae

Similarly, in the multivariate analysis when the left M.ES value of < 10% was taken as reference, in those with fat infiltration rate of 10–30%, the risk of LDH was 4.164-fold greater up to 14.097-fold (*p* = 0.022), and in those with 30%-50% fat infiltration, the risk was 36.99-fold greater increasing to 240.062-fold (*p* < 0.001). While the right M.ES was not significant in univariate analysis, when < 10% in multivariate analysis, in those with fat infiltration rate of 30%-50%, the risk of LDH was obtained as 0.110 (*p* = 0.007). The simplified 4-grade fat infiltration system predicted a strong relationship between fat infiltration and LDH (Table 4).

**Table 4:** The predictive strength of simplified 3 and 4-grade fat infiltration systems in lumbar disc herniation outcome

	Effect (strength) in predicting hernia
**Simplified 3-grade fat infiltration system**	
Grade 1: < 10% fat infiltration (normal)	nA
Grade2: 10–50% fat infiltration (moderate)	nA
Grade 3: < 50% fat infiltration (severe)	nA
**Simplified 4-grade fat infiltration system**	
Grade 1: < 10% fat infiltration (normal)	nA
Grade 2: 10–30% fat infiltration (mild)	5.806 (2.061 – 16.359) times
Grade 3: 30–50% fat infiltration (moderate)	36.99 (5.700 – 240.062) times
Grade 4: > 50% fat infiltration (severe)	nA

*nA* Not applicable

## Discussion

The main finding of this study was that the simplified 4-grade fat infiltration classification system was more effective than the simplified 3-grade system in the prediction of LDH. Different opinions have been stated about the fat infiltration of paraspinal muscles, which could be due to the weaknesses of different measurement methods. Therefore, there is a need for more sensitive measurement methods. In this study, a simplified 4-grade measurement method was formed and the sensitivityof this method was determined. There is no previous definition of a simplified 4-grade system in literature.

Different methods are used in the evaluation of fat infiltration. In the qualitative Goutallier method with the semi-quantitative simplified 3-grade system, inter and intra-observer reliability have been reported to be good [16, 26]. Kjaer et al. found inter and intra-observer reliability to be satisfactory but reported that visual evaluation of fat infiltration did not provide satisfactory results for adolescents [13]. While determining the degree of fat infiltration of the paraspinal muscles does not provide sufficient results by visual qualitative examination alone [4] semi-quantitative [13–16] and quantitative [17–24] measurements give satisfactory results.

In the simplified 3-grade system, when fat infiltration was assessed in the 10–50% band, no risk of LDH was revealed, whereas with fat infiltration in the 10–30% and 30–50% bands in the 4-grade system, a strong correlation was determined between fat infiltration and LDH. This result can be attributed to the very large breadth of the 10–50% band in the 3-grade system including the extreme values of 15% and 45%. Therefore, the separation of the 10–50% band into the more detailed 10–30% and 30–50% bands can be considered very important in reaching accurate results.

This can be explained as follows: The 10%-50% band in the 3-grade system included control group subjects with 15% fat infiltration and LDH patients with 45% fat infiltration, and no difference was found between the LDH group and control group in respect of fat infiltration. Therefore, the 3-grade system was thought to be insufficient for the prediction of LDH. However, when the same conditions were evaluated in the 4-grade system, healthy control group subjects with 15% fat infiltration were included in the 10%-30% band, and LDH patients with 45% fat infiltration were in the 30%-50% band. In this case there was a difference between the LDH patients and the control group, and this was able to predict LDH. The study was planned with the thought that the breadth of the 10%-50% was a deficiency of the 3-grade system. In the determination of fat infiltration > 50%, no error was observed as in the errors made in the determination of the fat infiltration rate in the 10%-50% band. However, as severe (> 50%) fat infiltration was not determined at a sufficient rate in either the LDH or control group, the groups could not be compared in respect of severe fat infiltration.

Fat infiltration seems to be a late stage of muscle degeneration, and fat infiltration of the lumbar musculus multifidus increases with increasing age in adults, irrespective of body composition [13]. Kidde et al. suggested that in respect of mobility function, muscle fat infiltration could be more important than muscle weakness [27]. Previous studies have stated a relationship between fat infiltration and lumbar disc herniation [11, 14, 15, 18, 25, 28–30]. In obese individuals, body fat accumulates naturally in the muscles along the muscle system of the back, and although spine problems are frequently seen, it does not settle at the level of the last two lumbar vertebrae. That fat infiltration is mainly found in these two problem areas tends to show that it is lower back pain that initiates muscle changes [4].

Muscle degeneration is characterised morphologically by muscle atrophy and increased fat accumulation [12, 31]. Similar to the findings of the current study, many studies have shown greater fat infiltrationn in individuals with low back pain compared to healthy subjects [13, 14, 26]. Conflicting results have been reported in literature on the subject of a relationship between fat infiltration and lumbar disc hernia. Some studies have reported a relationship between fat infiltration and only the m. multifidus [13, 31], some have shown a relationship with both the m.multifidus and the m. erector spina [13, 14, 31], and others have reported no relationship with either muscle [29, 32]. In extended review studies, the relationship between the paraspinal muscle morphology and chronic lumbar back pain has been shown to be uncertain. These studies have stated conflicting results for fat infiltration of the multifidus muscle and presented limited evidence for the erector spina muscle [25, 33, 34].

Limitations of the study can be considered to be the limited number of cases with > 50% fat infiltration, and that there are no other studies in literature with the same content for discussion of the 4-grade fat infiltration system.

## Conclusion

In conclusion, the results of this study demonstrated that the simplified 3-grade fat infiltration classification system could not predict LDH, whereas a strong correlation was determined with the 4-grade system. This shows the importance of using the semi-quantitative, simplified 4-grade fat infiltration classification system, which provides effective results in the prediction of the risk of LDH in individuals, rather than the simplified 3-grade fat infiltration system for the measurement of fat infiltration of muscles.

## Data Availability

The data obtained and analyzed in this study is not available to the public because of ethical regulations and local management procedures, but can be obtained on request from the corresponding author.

## Abbreviations

CT: Computed Tomography
MRI: Magnetic Resonance Imaging
US: Ultrasound
LDH: Lumbar Disc Herniation
M.MF: Musculus Multifidus
M.ES: Musculus Erector Spinae
